# Oxidative stress in sperm affects the epigenetic reprogramming in early embryonic development

**DOI:** 10.1186/s13072-018-0224-y

**Published:** 2018-10-17

**Authors:** Sarah Wyck, Carolina Herrera, Cristina E. Requena, Lilli Bittner, Petra Hajkova, Heinrich Bollwein, Raffaella Santoro

**Affiliations:** 1 Clinic of Reproductive Medicine, Department for Farm Animals, University of Zurich, 8057 Zurich, Switzerland; 2 Department of Molecular Mechanisms of Disease, DMMD, University of Zurich, 8057 Zurich, Switzerland; 3 Molecular Life Science Program, Life Science Zurich Graduate School, University of Zurich, 8057 Zurich, Switzerland; 4 MRC London Institute of Medical Sciences (LMS), London, W12 0NN UK; 5 Faculty of Medicine, Institute of Clinical Sciences (ICS), Imperial College London, London, W12 0NN UK

**Keywords:** Oxidative stress, Epigenetic reprogramming, BER, DNA methylation

## Abstract

**Background:**

Reactive oxygen species (ROS)-induced oxidative stress is well known to play a major role in male infertility. Sperm are sensitive to ROS damaging effects because as male germ cells form mature sperm they progressively lose the ability to repair DNA damage. However, how oxidative DNA lesions in sperm affect early embryonic development remains elusive.

**Results:**

Using cattle as model, we show that fertilization using sperm exposed to oxidative stress caused a major developmental arrest at the time of embryonic genome activation. The levels of DNA damage response did not directly correlate with the degree of developmental defects. The early cellular response for DNA damage, γH2AX, is already present at high levels in zygotes that progress normally in development and did not significantly increase at the paternal genome containing oxidative DNA lesions. Moreover, XRCC1, a factor implicated in the last step of base excision repair (BER) pathway, was recruited to the damaged paternal genome, indicating that the maternal BER machinery can repair these DNA lesions induced in sperm. Remarkably, the paternal genome with oxidative DNA lesions showed an impairment of zygotic active DNA demethylation, a process that previous studies linked to BER. Quantitative immunofluorescence analysis and ultrasensitive LC–MS-based measurements revealed that oxidative DNA lesions in sperm impair active DNA demethylation at paternal pronuclei, without affecting 5-hydroxymethylcytosine (5hmC), a 5-methylcytosine modification that has been implicated in paternal active DNA demethylation in mouse zygotes. Thus, other 5hmC-independent processes are implicated in active DNA demethylation in bovine embryos. The recruitment of XRCC1 to damaged paternal pronuclei indicates that oxidative DNA lesions drive BER to repair DNA at the expense of DNA demethylation. Finally, this study highlighted striking differences in DNA methylation dynamics between bovine and mouse zygotes that will facilitate the understanding of the dynamics of DNA methylation in early development.

**Conclusions:**

The data demonstrate that oxidative stress in sperm has an impact not only on DNA integrity but also on the dynamics of epigenetic reprogramming, which may harm the paternal genetic and epigenetic contribution to the developing embryo and affect embryo development and embryo quality.

**Electronic supplementary material:**

The online version of this article (10.1186/s13072-018-0224-y) contains supplementary material, which is available to authorized users.

## Background

Infertility affects around 15% of all couples of reproductive age, with about 50% being associated with abnormalities in the male [3, 73]. Most of the cases of male infertility are caused by abnormal spermatogenesis and failure in sperm function. A decrease in male fertility has been associated with environmental factors (i.e. exposure to certain chemicals, heavy metals, pesticides and heat), smoking, alcohol abuse, chronic stress, obesity, urogenital trauma and inflammation in the male reproductive system [20, 26, 64].

Reactive oxygen species (ROS)-induced oxidative stress is well known to play a major role in male factor infertility [70]. Oxidative stress occurs when the production of potentially destructive ROS exceeds the body’s own natural antioxidant defences, resulting in cellular damage. Oxygen is important for the aerobic metabolism of spermatogenic cells [57, 64]. In physiological amounts, ROS are essential requirements of spermatozoa for sperm processes that lead to successful fertilization, such as capacitation, hyperactivated motility and acrosomal reaction [5, 16]. However, sperm are particularly susceptible to the damaging effects of ROS since their cell membrane is composed of large amounts of unsaturated fatty acids, which can be oxidized, and contain few amounts of scavenging enzymes able to neutralize ROS [13, 15]. These factors can affect membrane integrity, motility as well as the ability to fertilize oocytes [4, 6]. Of the four DNA bases, guanine is the most susceptible to oxidation and the most common oxidative DNA lesions is 8-oxoguanine (8-oxoG), which is highly mutagenic. 8-oxoG is repaired by 8-oxoguanine DNA glycosylase-1 (OGG1) during the DNA base excision repair pathway (BER) [48]. Furthermore, DNA fragmentation may harm the paternal genetic contribution to the developing embryo [70]. The post-meiotic phase of mouse spermatogenesis is very sensitive to the genomic effects of environmental mutagens because as soon as male germ cells form mature sperm they progressively lose the ability to repair DNA damage, harming the paternal genetic contribution to the developing embryo [47, 52]. Consequentially, it is believed that oxidative DNA damage in sperm can be repaired only post-fertilization by the maternal BER machinery [44]. However, extensive DNA damage in sperm can exceed the maternal repair capacities and have a direct impact on subsequent development [46, 61]. To this point, how oxidative stress in sperm affects early development is not fully understood.

DNA methylation is a crucial element in the epigenetic regulation of mammalian embryonic development [51]. After fertilization, the two specialized and highly differentiated cells, the oocyte and the sperm, fuse to form the zygote. In order to reset the gamete’s epigenome into a totipotent state, both parental and maternal genomes undergo epigenetic reprogramming. In early embryos, DNA methylation is reprogrammed genome-wide. Shortly after zygote formation, the mature sperm genome is globally demethylated, with exception of a limited number of loci including parental imprints and active retrotransposons, which are protected from demethylation to ensure embryonic viability [50, 53, 62].

It has been proposed that loss of DNA methylation at paternal genome is mediated by active DNA demethylation mechanisms as it occurs before the onset of DNA replication [50, 53]. Conversely, the maternal genome undergoes replication-dependent DNA demethylation (passive demethylation), further adding to a parental epigenetic asymmetry in the zygote. Similar DNA demethylation pattern was detected in several other mammals (i.e. human, mouse, rat and cattle), whereas in other species, such as pigs and goats, DNA demethylation is still controversial [18, 24, 35, 54, 56]. The mechanism of active DNA demethylation utilized in zygotes is poorly understood.

Active DNA demethylation has been proposed to be a multistep process that is initiated by modifications of the methylated cytosine or methyl group, followed by replication-based dilution or removal of the modified base via a DNA repair mechanism. In the mouse zygotes, pharmacological inactivation of components of the BER pathway resulted in zygotes with significantly higher levels of paternal DNA methylation, suggesting that BER might play an important role in active DNA demethylation [32, 79]. A pathway recently suggested for active DNA demethylation in the early mouse embryo involves the conversion of 5-methylcytosine (5mC) to 5-hydroxymethylcytosine (5hmC) mediated by TET3, a member of the ten-eleven translocation (Tet) family of DNA dioxygenases that is expressed at high levels in oocytes and zygotes [36]. In the mouse zygotes, the paternal pronucleus (pPN) contains substantial amounts of 5hmC but lacks 5mC; furthermore, the depletion of *Tet3* affects both 5hmC and 5mC patterns [28, 36, 78].

In this study, we set out to analyse how oxidative stress affects early embryo development using the bovine system due to its similarity to early human embryo development [60, 65]. Fertilization using sperm exposed to oxidative stress caused a major developmental arrest at the time of embryonic genome activation. Remarkably, the DNA demethylation of paternal genome harbouring oxidative lesions was impaired. The recruitment of XRCC1, a factor involved in the final step of BER pathway, to the paternal genome containing oxidative DNA lesions indicates that the zygotic BER pathway recognizes and repairs DNA lesions at the expense of DNA demethylation. The impairment of active DNA demethylation did not affect 5hmC levels in zygotes, indicating that other 5hmC-independent processes are implicated in active DNA demethylation in bovine embryos. Together, our study demonstrates that next to the impact on DNA integrity, oxidative stress in sperm has a direct effect on the dynamics of epigenetic reprogramming. This in turn may harm the paternal genetic and epigenetic contribution to the developing embryo and affect embryo development and embryo quality. Last but not least, our results reveal species-specific epigenetic differences between bovine and mouse embryos and gametes that will facilitate the understanding of the dynamics of DNA methylation in early development.

## Results

### Oxidative stress in sperm affects early embryonic development

**Fig. 1:**
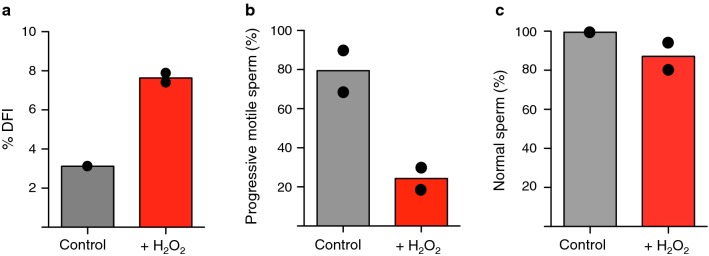
Oxidative stress reduces motility and increases DNA damage in sperm. **a** Sperm chromatin structure measurements (%DFI) of sperm treated without (control) and with H_2_O_2_ (+H_2_O_2_) were performed using the sperm chromatin structure assay (SCSA™). Mean and values of two independent experiments are shown. Progressive motility (**b**) and morphology (**c**) of sperm treated without and with H_2_O_2_ were analysed by computer-assisted sperm analysis (CASA). Mean and values of two independent experiments are shown

**Fig. 2:**
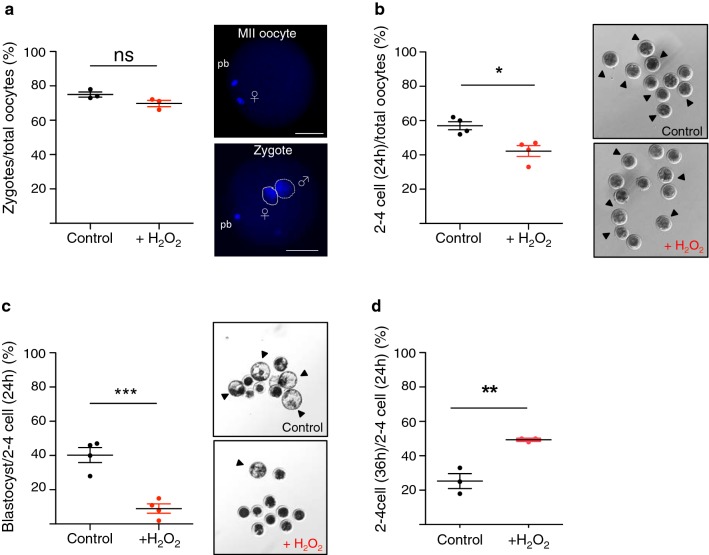
The developmental capacity of embryos is drastically reduced upon oxidative stress in sperm. **a** Fertilization rates of sperm treated without (control) or with H_2_O_2_ (+H_2_O_2_) were evaluated 20 h after IVF by staining the pronuclei with DAPI. Images were acquired with an inverted Leica CTR6000 microscope (software: Leica Microsystems LAS-AF6000; Leica Microsystems, Bensheim, Germany). The fertilization capacity (%) was quantified by calculating the number of obtained zygotes versus the total number of oocytes used in each IVF experiment. Each data point represents the mean of one of four independent experiments. Total amount of oocytes: 225 (control) and 209 (+H_2_O_2_). **b** Cleavage rate (%) was quantified by calculating the number of cleaved embryos (two-cell stage and further) obtained 24 h after IVF versus the total number of oocytes used in each IVF experiment. Each data point represents the mean of one of the four independent experiments. Total amount of oocytes: 100 (control), 123 (+H_2_O_2_). Arrows indicate the cleaved embryo stages. **c** Blastocyst rate (%) was quantified by calculating the number of blastocysts obtained 8 days after IVF versus the number of embryos that reached the cleavage stage 24 h after IVF. Each data point represents the mean of one of the four independent experiments. Total amount of oocytes: 148 (control) and 165 (+H_2_O_2_). Arrows indicate the blastocyst stage embryos. **d** Arrested embryos were evaluated 36 h after IVF. Quantifications were assessed by counting the number of embryos that were arrested at two- to four-cell stages 36 h after IVF versus the number of embryos that already reached two- to four-cell stage 24 h after IVF. Each data point represents the mean of one of three independent experiments. Total amount of oocytes: 127 (control) and 141 (+H_2_O_2_). Statistical analyses were performed using Student’s *t*-test (two-tailed). Error bars indicate s.d. ns: non-significant and refers to *P *= 0.091; *P < 0.05; **P < 0.01; ***P < 0.0001. Maternal pronucleus: mPN/♀/continuous line; paternal pronucleus: pPN/♂/dashed line. Scale bars, 50 μm

To determine whether and how oxidative stress in sperm affects early embryonic development, we aimed to use conditions that induce DNA damage in sperm without harming its fertilization capacity using in vitro fertilization (IVF). We treated cryopreserved sperm of a fertile bull from an approved artificial insemination (AI) station with 100 μm H_2_O_2_ for 1 h and analysed the effects of this treatment on sperm motility, morphology and DNA integrity. Higher concentrations of H_2_O_2_ induced cell death (data not shown). We performed sperm chromatin structure assay (SCSA™), which yields information on strand breaks but also reveals the presence of DNA adducts or abasic sites [66]. As expected, the percentage of sperm with a high DNA fragmentation index (%DFI) significantly increased upon H_2_O_2_ treatment (control: 3.1%; H_2_O_2_: 7.6%) (Fig. 1a). Such an increase in %DFI for a fertile bull from an AI station is generally considered to lead to an impairment of fertility [22, 37]. The increase in %DFI in sperm exposed to oxidative stress was consistent with previous studies showing that OGG1 is active in sperm as it can produce abasic sites at oxidative DNA lesions. However, these abasic sites cannot be repaired in sperm due to the lack of AP endonucleases and XRCC1 [44, 66]. Next, we measured the effects of oxidative stress on progressive motility and morphology by performing computer-assisted sperm analysis (CASA) that is routinely used in sperm quality control. Progressive motility of the sperm was reduced in the group treated with H_2_O_2_ (control: 79.5%; H_2_O_2_: 24.2%) (Fig. 1b). In contrast, the overall morphology was not greatly affected upon H_2_O_2_ treatment (control: 99.5%; H_2_O_2_: 87.1%) (Fig. 1c). Finally, to determine whether these oxidative stress conditions affect the penetration of sperm into the oocyte, we measured the fertilization rate by performing IVF using slaughterhouse oocytes and H_2_O_2_-treated and untreated bovine sperm. After H_2_O_2_ treatment, sperm were extensively washed to ensure that the oxidative stress was only limited to sperm itself without affecting oocyte integrity. Fertilization rate was measured by staining the pronuclei of the presumptive zygotes with DAPI and quantifying the number of obtained zygotes relative to the total number of oocytes used for each IVF cycle (Fig. 2a). Using these conditions, we did not observe remarkable changes in the fertilization rate (control: 75.0 ± 1.5%; H_2_O_2_: 69.0 ± 1.9%) (Fig. 2a). These results indicate that the conditions used for oxidative stress induce DNA damage without impacting the fertilization capacity of the sperm.

To determine whether and how oxidative stress in sperm affects the early steps of embryo development, we performed IVF and measured cleavage rate and blastocyst formation of embryos derived from sperm of control and H_2_O_2_-treated groups. A first critical checkpoint in early embryonic development is the capability of the growing embryos to undergo the first cell divisions. We quantified the first cell division of embryos 24 h after IVF by calculating the number of cleaved embryos (two-cell stage and further) versus the total number of oocytes used in each IVF experiment (Fig. 2b). In control samples, we obtained a cleavage rate of 57.0 ± 2.4%, whereas the cleavage rate after IVF with sperm treated with H_2_O_2_ was 42.3 ± 3.2%, indicating that oxidative stress in sperm induces some defects in this early stage of development.

Next, we analysed the impact of oxidative stress in sperm on further developmental progression. Blastocyst formation was measured by calculating the number of blastocysts obtained 8 days after IVF versus the number of cleaved embryos obtained 24 h after IVF (two-cell stage and further) (Fig. 2c). Blastocyst formation in the control group was 40.3 ± 4.4%. Remarkably, the number of blastocysts originated from fertilization with sperm treated with H_2_O_2_ was drastically reduced (9.0 ± 2.7%). These results indicate that H_2_O_2_ treatment of sperm induces major defects after the first cleavage. Embryonic genome activation (EGA) in bovine embryos starts at four-cell stage, and this is considered another important checkpoint of early embryonic development [27]. Therefore, we asked whether embryos fertilized with H_2_O_2_-treated sperm were arrested at this time point. We monitored the number of embryos of control and H_2_O_2_ groups 36 h after fertilization and found that 49.3 ± 0.7% of embryos derived from fertilization with sperm treated with H_2_O_2_ were still at two- or four-cell stages while the majority of embryos of the control group reached further developmental stages (Fig. 2d).

Taken together, these results indicate that oxidative stress in sperm affects early embryonic development and that the major defects occur in later phase of development, closed to embryonic genome activation.

### The BER machinery is recruited to the paternal genome of zygotes generated from sperm harbouring oxidative DNA lesions

**Fig. 3:**
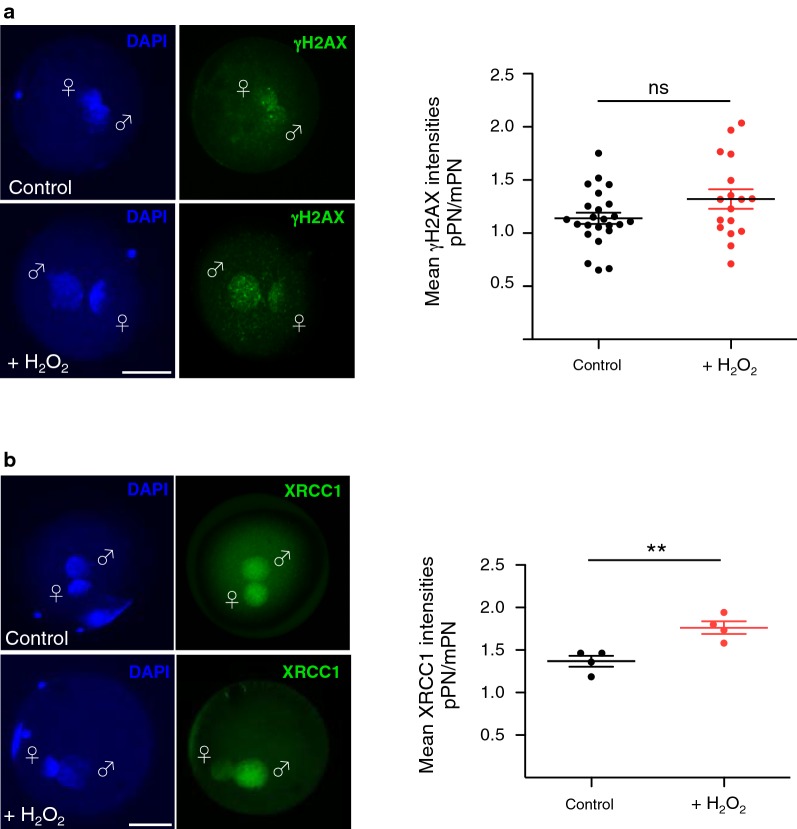
The BER machinery is sequestered to paternal pronuclei of zygotes derived from sperm exposed to oxidative stress. **a** γH2AX (green) levels were assessed by immunofluorescence of zygotes 20 h after IVF using γH2AX antibodies. Representative images of PN3/4 zygotes are shown. Quantification of γH2AX is represented as a ratio of the mean signal intensity between the two pronuclei signal (mean γH2AX intensities pPN/mPN). (*n *= 24 zygotes control and *n *= 17 H_2_O_2_-treated zygotes; experiment replicated three times independently.) Each data point represents the mean signal of the ratio (pPN/mPN) within an independent zygote. **b** Representative immunofluorescence images of PN3/4 zygotes 20 h after IVF stained with XRCC1 antibody (green) are shown. Quantifications of XRCC1 are shown as ratio of the mean signal intensity between the two pronuclei signal (pPN/mPN). Each data point represents the value of four independent experiments, using 120 zygotes for the control group and 85 zygotes from +H_2_O_2_ groups. Statistical analysis was performed using Student’s *t*-test (two-tailed). Error bars indicate s.d.; **P < 0.01; ns: non-significant and refers to *P *= 0.075. Maternal pronucleus: mPN/♀; paternal pronucleus: pPN/♂. Scale bars, 50 μm

To determine the extent of DNA damage at the paternal pronucleus of zygotes generated with sperm harbouring oxidative DNA lesions, we measured γH2AX, an early cellular response to the induction of DNA double- and single-strand breaks [69]. We observed that γH2AX was abundantly present at both maternal and paternal pronuclei of the control zygotes, which, however, progress normally through early development (Fig. 3a). Surprisingly, we did not observe any enrichment of γH2AX at paternal pronuclei compared with maternal pronuclei, which is in contrast to the higher γH2AX signals before and during replication of paternal pronuclei described in mouse zygotes [79, 81]. Furthermore, although the set-up of this experiment did not allow us to distinguish bovine zygotes in a pre-replicative state or during S phase, we did not detect a significant increase in γH2AX signal at paternal genome that underwent oxidative damage prior fertilization. These results indicated that the levels of γH2AX induced by oxidative stress in sperm did not directly correlate with the arrest in early embryo development observed in embryo obtained with sperm containing oxidative DNA lesions.

BER is the major cellular repair pathway responsible for repair of the oxidative DNA lesions [48]. Since all major DNA repair pathways are less functional in late spermatids and sperm [47], we asked whether the maternal BER machinery in zygotes has the potential to repair the paternal genome harbouring oxidative DNA lesions. We measured the localization of XRCC1 (X-ray repair cross-complementing protein (1), a factor involved in the final step of BER pathway that serves as scaffold for DNA ligases in the ligation of the DNA strand nick [49, 72] (Fig. 3b). In control zygotes, XRCC1 was equally distributed between paternal and maternal pronuclei. In contrast, in zygotes obtained with sperm that underwent oxidative stress, a large portion of XRCC1 was recruited to the paternal pronucleus. These results indicated that oxidative lesions induced in the sperm prior to fertilization can be repaired in zygotes by the BER pathway. Together, these data suggest that oxidative DNA damage response cannot be the only reason for embryo developmental arrest since zygotes that progress normally in development already contain high level of DNA damage, the additive γH2AX induced by oxidative stress in sperm is barely detectable and zygotes have the potential to repair these oxidative DNA lesions through BER pathway.

### Oxidative stress in sperm impairs active DNA demethylation in the paternal pronucleus

The results described above implicated the BER pathway in the repair of oxidative DNA lesions of the paternal genome in zygotes. However, the BER pathway has also been implicated in the active DNA demethylation of the paternal genome in the mouse zygotes [32, 77]. Pharmacological inactivation of the BER core components—APE1 (apurinic/apyrimidinic endonuclease) and PARP1 (poly(ADP-ribose) polymerase family, member (1)—resulted in zygotes with significantly higher levels of DNA methylation in the paternal pronucleus [32]. Moreover, XRCC1 was shown to have a pronounced chromatin association in the paternal pronucleus of mouse zygotes [32]. The implication of BER in both DNA repair and active DNA demethylation activities at the paternal genome prompted us to ask whether the paternal genome containing DNA lesions could be efficiently DNA demethylated.

**Fig. 4:**
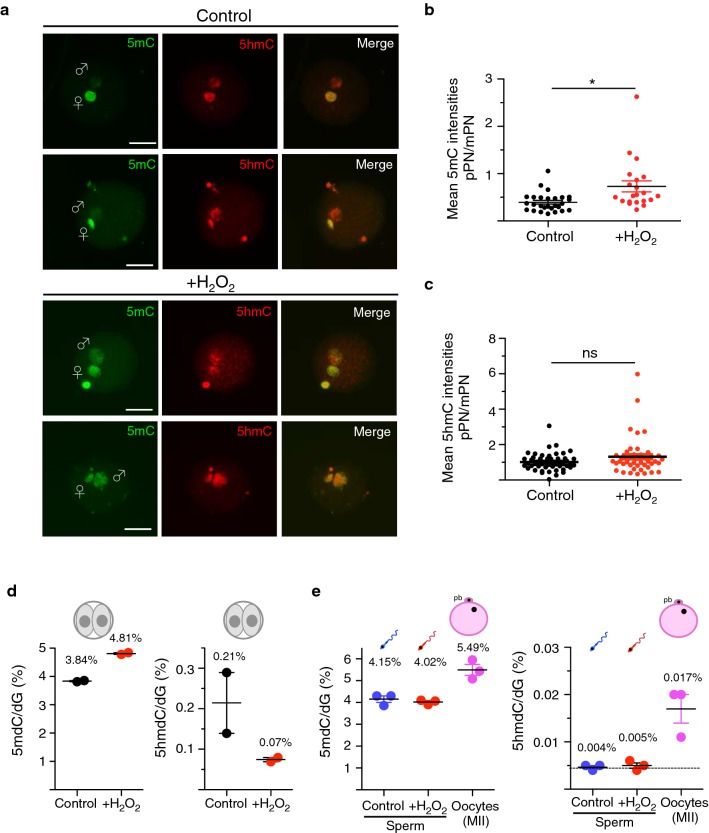
Oxidative stress in sperm impairs active DNA demethylation on the paternal pronucleus. **a** Representative immunofluorescence images showing the levels of 5mC (green) and 5hmC (red) in PN3/4 zygotes using 5mC- and 5hmC-specific antibodies. Zygotes were analysed 20 h after IVF. Quantifications of 5mC (**b**) and 5hmC (**c**) signals are shown as ratio of the signal of the mean intensity of the paternal pronucleus (pPN) over the mean intensity signal of the maternal pronucleus (mPN) after background subtraction. Each data point represents an independent zygote (5mC control n = 27 zygotes and 5mC +H_2_O_2_ = 21 zygotes, three independent experiments; 5hmC control *n * = 52 and 5hmC +H_2_O_2_ n = 48, three independent experiments). Statistical analysis was performed using Student’s *t*-test (two-tailed). Error bars indicate s.d., ns: non-significant **P < 0.01. Maternal pronucleus: mPN/♀; paternal pronucleus: pPN/♂. Scale bars, 50 μm. **d** Quantification of 5mC/dG by LC–MS of two-cell embryos obtained upon IVF with control sperm and sperm treated with H_2_O_2_. Data are from two independent experiments. Each data point represents the mean of two technical replicates of a pool of 50 embryos. **e** Quantification of 5mC and 5hmC by LC–MS of bovine sperm untreated or treated with H_2_O_2_ and MII oocytes. Data are from three independent experiments. Each data point represents the mean of two technical replicates

To test whether oxidative stress in sperm affects DNA demethylation, we measured 5mC in bovine zygotes by immunofluorescence (IF) using anti-5mC-specific antibodies. 24 h after IVF, most of the pronuclei in the zygotes reached late pronuclear stage 3 or 4 (PN3/4). The size of the two pronuclei was informative of the parental origin: the paternal pronucleus (pPN) displays a more enlarged structure, which reflects the level of chromatin decondensation, compared with the maternal pronucleus (mPN). As shown in Fig. 4a, b, 24 h post-IVF the pPN in control zygotes shows a dramatically reduced 5mC signal compared with the mPN, which still retained DNA methylation. These results are consistent with previous reports, showing that also the paternal genome in bovine embryos undergoes DNA demethylation [17, 42, 78]. Remarkably, zygotes fertilized with sperm treated with H_2_O_2_ retained higher levels of DNA methylation, indicating that the active DNA demethylation process in the pPN is compromised (Fig. 4a, b). The pPN volume of H_2_O_2_-treated group was similar to the control, suggesting that impairment of DNA demethylation was not due to defects in chromatin decondensation (Additional file 1: Fig. S1). In mice, DNA demethylation of the paternal genome is accompanied by the establishment of cytosine hydroxymethylation (5hmC), a reaction that has been implicated in the loss of DNA methylation [41, 67]. To test whether the impairment of DNA demethylation correlated with changes in 5hmC levels in the paternal pronuclei, we analysed 5hmC content by IF using specific 5hmC antibodies. Interestingly, IF analyses revealed that both paternal and maternal pronuclei at PN3/4 contain equal amounts of 5hmC (average mean intensity of 5hmC pPN versus mPN equal to 1.01, Fig. 4a, c). This pattern observed in bovine zygotes is similar to the 5hmC localization observed in human zygotes but differs from what has been described in mouse zygotes where 5hmC is present only in the pPN [30, 79]. These results indicated that 5hmC in bovine zygotes as well as in human is not only restricted to the paternal genome. Remarkably, 5hmC levels were not considerably affected in zygotes obtained with sperm containing oxidative DNA lesions, indicating that oxidative stress in sperm impairs DNA demethylation without altering global 5hmC levels (Fig. 4a, c). Recent results have revealed that in mouse zygotes the loss of paternal 5mC and accumulation of 5hmC are temporally disconnected, with a first wave of DNA demethylation that is independent of 5hmC followed by zygotic de novo DNA methylation activities that trigger TET3-driven methylcytosine hydroxylation [9]. Thus, our results showing that oxidative stress in sperm impairs active DNA demethylation without altering 5hmC suggest that these paternal oxidative DNA lesions impair the first, 5hmC-independent wave of active DNA demethylation. It is also important to note that 5mC and 5hmC signals cannot be directly compared since 5hmC antibody shows about 10,000-fold greater sensitivity than the 5mC antibody [9]. To provide quantitative measurements and to further support our results obtained by IF analyses, we quantified DNA methylation content of isolated two-cell stage embryos using liquid chromatography–tandem mass spectrometry (LC–MS/MS), a highly quantitative method to measure DNA methylation (Fig. 4d). Consistent with the IF analysis in zygotes, two-cell stage embryos obtained using H_2_O_2_-treated sperm retained higher 5mC levels than control embryos (Fig. 4a, b). Importantly, 5mC content was similar in control and H_2_O_2_-treated sperm, indicating that oxidative stress has no impact on DNA methylation prior to fertilization (Fig. 4e). These measurements also showed that the majority of cytosine hydroxylation occurs after fertilization as 5hmC levels were higher in two-cell embryos than in sperm and oocyte. Interestingly, 5hmC was lower in two-cell stage embryos generated with H_2_O_2_-treated sperm compared with control embryos (0.21 vs. 0.07%) (Fig. 4d). Considering that our IF measurements did not detect any changes in 5hmC levels in zygotes, these results suggest that oxidative stress in sperm might also have an effect on methylcytosine hydroxylation activities during/after the first zygotic replication. Accordingly, recent genome-scale DNA methylation maps for both the paternal and maternal genomes suggested that TET3 might facilitate DNA demethylation largely by coupling with DNA replication [63]. Moreover, an increase in 5hmC that was unrelated to any change in 5mC level was also observed after the completion of zygote replication [58]. Together, these results indicated that oxidative DNA lesions in sperm impair active DNA demethylation of the paternal genome in zygotes, leading to alterations in the epigenetic reprogramming during early development. Moreover, the data highlighted substantial differences in the dynamic of cytosine hydroxymethylation between species, showing higher similarity between bovine and human embryos with respect to mouse embryos.

### Unmodified cytosines are incorporated in pre-replicative bovine zygotes

**Fig. 5:**
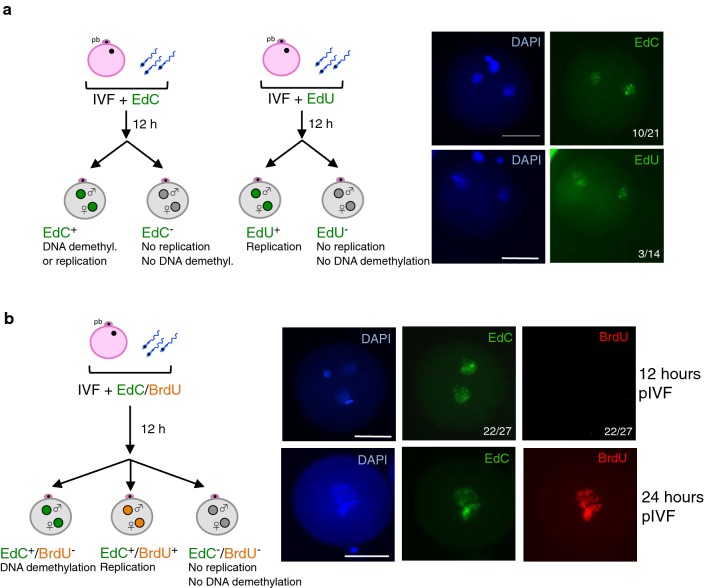
Replacement of cytosines in pre-replicative zygotes. **a** Schema describes the strategy to measure replication and DNA demethylation via the incorporation of EdU and EdC in zygotes and its readout. Representative images showing zygotes with EdU (green) and EdC (green) incorporation 12 h after IVF. Numbers refer to zygotes showing EdU or EdC incorporation relative to the total number of analysed zygotes, respectively. **b** Schema describes the strategy to measure replication and DNA demethylation via the incorporation of BrdU and EdC in zygotes and its readout. Representative images showing zygotes stained for BrdU (red) and EdC (green) incorporation 12 h after IVF. Numbers refer to zygotes positive for EdC signal and negative for BrdU signal relative to the total number of analysed zygotes. Zygotes 24 h pIVF were stained and used as post-replicative control zygotes. Scale bars, 50 μm

The results described above showed that the pattern of γH2AX and 5hmC differs between bovine and mouse zygotes. Both modifications have been linked to active DNA demethylation in zygote and found to be enriched at paternal pronuclei compared with maternal pronuclei of mouse zygotes whereas in bovine zygotes are present at similar levels at both pronuclei (Figs. 3a, 4a, c). The presence of γH2AX at mouse paternal genome has led to suggest the involvement of a repair-coupled pathway in active DNA demethylation since the replacement of 5mC or its derivatives with unmodified cytosine should create DNA strand breaks and as such it should induce DNA damage response including γH2AX [77]. The detection of 5hmC and γH2AX at both pronuclei of bovine zygotes raised the question whether also the maternal genome can undergo some minor waves of active DNA demethylation. To do so, we analysed the incorporation of unmodified cytosines into the paternal and maternal pronuclei of pre-replicative zygotes by performing IVF in the presence of nucleotide analogues (EdU or EdC) and monitored their incorporation by click chemistry 12 h after IVF (Fig. 5a). The incorporation of EdU will be indicative of DNA replication, whereas the incorporation of EdC will correspond to either DNA replication or the replacement of an unmodified cytosine. Our pilot experiments indicated that 12 h after IVF the majority of zygotes is in a pre-replicative state. Accordingly, only few zygotes (three out of 14) incorporated EdU, indicating that only a small number of zygotes have already entered S phase. In contrast, ten out of 21 analysed zygotes incorporated EdC, suggesting that unmodified cytosines are incorporated at both pronuclei in the absence of replication. To further support these results, IVF was performed in the presence of BrdU and EdC and 12 h after IVF their incorporation was measured by double staining using anti-BrdU and click chemistry for EdC (Fig. 5b). As control, we analysed zygotes that had already accomplished the first cell cycle (24 h after IVF) and were therefore positive for both BrdU and EdC. None of the zygotes analysed 12 h after IVF incorporated BrdU, indicating a pre-replicative state at this time point. Remarkably, 22 out of the 27 analysed zygotes showed EdC signals at both paternal and maternal pronuclei. These two different experimental approaches unequivocally demonstrated that cytosines are incorporated into the DNA of pre-replicative zygotes and further support a mechanism by which active DNA demethylation might occur via excision of 5mC or its oxidative forms followed by replacement with unmodified cytosines. Furthermore, the incorporation of unmodified cytosines at both paternal and maternal pronuclei in pre-replicative bovine zygotes is consistent with the presence of 5hmC and γH2AX at both pronuclei and suggests the occurrence of some zygotic DNA demethylation activities also at maternal genome. Locus-specific active DNA demethylation of the maternal genome was already described in mouse zygotes [29, 75]. Our results thus raise the possibility that also in bovine embryos active DNA demethylation might not be exclusively restricted to the paternal genome.

## Discussion

DNA damage in the male germ line is mainly caused by the presence of unbalanced reactive oxygen species, which might contribute to infertility, miscarriage and birth defects in the offspring [7]. In this study, we evaluated how oxidative stress in sperm affects early embryo development using conditions that induced DNA damage without affecting the fertilization rate. Enhanced recruitment of the BER core component XRCC1 at pPN of zygotes generated with sperm exposed to oxidative stress indicates that the BER pathway is implicated in this zygotic DNA damage response. It has been suggested that early-stage embryos have a different DNA damage response compared to somatic cells, which normally activate cell cycle checkpoints [1, 25, 71]. Accordingly, the major defects of embryos obtained with H_2_O_2_-treated sperm were identified in later time points of development—at two- to four-cell stages—with a 78% reduction in the formation of blastocysts from embryos that progress beyond the 2-cell stage. Similar observations have been described in previous studies in cattle and primates, showing that DNA fragmentation has an impact in later phase of development [11, 14, 23, 68].

Why does oxidative stress in sperm induce an arrest in early embryo development only after the first cell division, at the two- to four-cell stage? We can envision several possible scenarios. In the first case, the oxidative DNA lesions present in the paternal genome cannot be properly repaired in the zygotes and consequently embryonic DNA damage response can promote, for yet unknown reasons, cell cycle arrest only after the first cell division. However, the fact that the BER machinery was recruited to damaged pPN indicates that zygotes can repair, at least in part, these lesions. Moreover, the presence of high γH2AX signal at both paternal and maternal genome of control zygotes and the lack of any evident increase of γH2AX at paternal pronuclei upon oxidative stress in sperm suggest that oxidative DNA lesions alone cannot be responsible for embryo developmental arrest. Although we cannot exclude that sperm exposed to an oxidative environment may carry toxic metabolites that would then impair embryo development, it is notable that the major embryonic arrest occurs at the onset of bovine embryonic genome activation. Thus, a possible further explanation is that the impairment of active DNA demethylation at paternal genome might affect the expression of genes critical to development due to failure in zygotic epigenetic reprogramming.

**Fig. 6:**
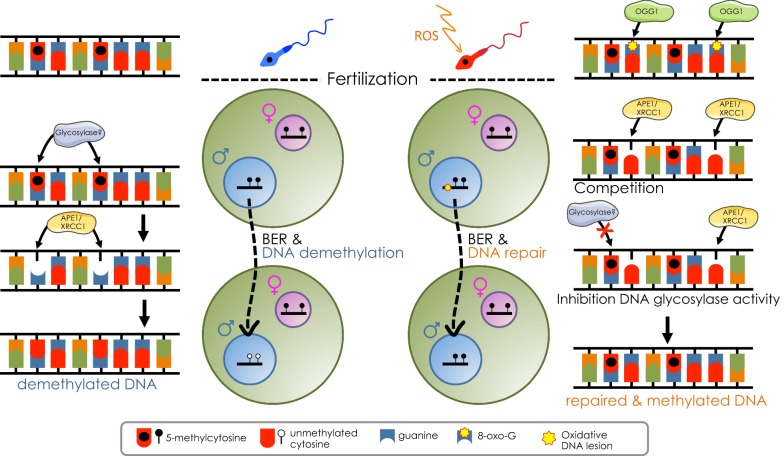
Oxidative lesions in sperm impair active DNA demethylation at the paternal genome in zygotes. The model shows the link of BER to DNA damage and active DNA demethylation. On the left, it is shown how a putative DNA glycosylase recognizes 5mC or its modified forms, giving rise to abasic sites that via the subsequent enzymes of the BER pathway (i.e. APE1 and XRCC1) allows the incorporation of unmodified cytosines. On the right, it is shown how oxidative lesions in sperm can impair DNA demethylation. Two models are here shown. In the first case, the presence of abasic sites that are established by OGG1 prior fertilization in sperm sequester XRCC1 at the expense of DNA demethylation activities, where recognition and excision of 5mC or its derivative is initiated only post-fertilization. The second model suggests that DNA lesions inhibit the activity of the DNA glycosylase(s) responsible for the DNA demethylation. In both cases, the final product is a DNA that is repaired but still contains methylated cytosines

The finding that active DNA demethylation is impaired in zygotes obtained with sperm exposed to oxidative damage provides a strong indication that this process is likely linked to DNA repair pathways. Moreover, we provide direct evidence that cytosines are incorporated into pre-replicative zygotes, further supporting a mechanism by which DNA demethylation might occur via excision of 5mC or its oxidative forms and replacement with unmodified cytosines. The accumulation of BER components at pPN of zygotes originating from sperm with oxidative DNA lesions suggests a competition model between repair and DNA demethylation activities, where oxidative lesions at paternal DNA are repaired at the expense of DNA demethylation (Fig. 6). A switch in BER activity that favours the repair of oxidative lesions (e.g. 8-oxoG) over the replacement of 5mC or 5mC oxidized products can be explained by the different activities between DNA glycosylases, which recognize specific base modifications and generate an abasic site product [38]. OGG1 is the glycosylase implicated in the repair of oxidized DNA lesions. Interestingly, OGG1 has been identified in the chromatin of human spermatozoa [44, 66]. OGG1 can cleave 8-oxoG adducts from sperm nuclear DNA to create the corresponding abasic sites that, however, cannot be repaired in sperm due to the lack of AP endonucleases and XRCC1. Thus, in the zygote, the presence of already established abasic sites at pPN might explain the sequestration of BER components such as XRCC1 at the expense of DNA demethylation activities, where recognition and excision of 5mC or its derivative is initiated only post-fertilization. Another possibility is that DNA lesions inhibit the activity of the DNA glycosylase(s) responsible for the DNA demethylation (Fig. 6). Thymine DNA glycosylase (TDG) was implicated in DNA demethylation due to its ability to recognize and remove the oxidized forms of 5mC, 5fC and 5caC [33, 39, 45]. In vitro biochemical analyses indicated that the removal of symmetrically methylated CpGs by TDG/TET1 complex occurs in a sequential manner and that the presence of the DNA lesions on one strand delays the nucleotide removal of the opposite strand [76]. Although this can represent a possible scenario occurring at the paternal genome harbouring DNA lesions in the zygote, the factors implicated in DNA demethylation in the zygotes remain still elusive. Indeed, TET1 is present only in later stages of early embryonic development and *Tdg* deletion from the zygote has no effect on DNA demethylation [29, 33, 39]. Thus, the identification of glycosylases implicated in the excision of 5mC or its modified derivatives will be important in order to understand not only the dynamics of DNA demethylation but also the impact of DNA damage on the early embryo epigenetic reprogramming. Finally, our results excluded the possibility that DNA damage might affect protamine–histone exchange and chromatin decondensation of the paternal genome, which have been thought to be necessary for active DNA demethylation [43, 55], as we did not observe any apparent defects in decondensation of paternal pronuclei as well as the incorporation of the early embryo epigenetic mark H3K27me3 [59] (Additional file 1: Fig. S1 and data not shown).

Our study also revealed several differences between bovine and mouse embryos. Although DNA demethylation of the paternal genome was conserved, factors previously linked to active DNA demethylation in the mouse zygotes, namely γH2AX and 5hmC, displayed a distinct localization [36, 77, 78]. In the mouse zygotes, γH2AX and 5hmC were shown to be enriched in the pPN, whereas in bovine, they were equally present at both paternal and maternal pronuclei. Interestingly, a recent study showed that 5hmC in human embryos is also localized at both paternal and maternal pronuclei [30]. Thus, the similar 5hmC pattern between bovine and human embryos makes the bovine model an interesting system to understand the epigenetic remodelling in human embryo, which for ethical issues cannot be easily studied. Remarkably, the impairment of active DNA demethylation in bovine zygotes obtained with sperm exposed to oxidative stress was not accompanied by alterations in 5hmC content. These results indicated that the active DNA demethylation in bovine embryos might not completely depend on the 5hmC pathway. Remarkably, recent studies using mouse models showed that in the zygotes the loss of paternal 5mC and accumulation of 5hmC are temporally disconnected and proposed that TET3 might play a major role in preventing aberrant de novo methylation from the abundant DNMT3A inherited from the oocyte [9]. Along the same lines, it was recently shown that in the gonadal mouse primordial germ cells 5hmC was not a prerequisite for the 5mC loss and that TET1 played a role in maintaining but not driving DNA demethylation [34]. Thus, the detection of 5hmC in the bovine paternal pronuclei with impaired DNA demethylation suggests that H_2_O_2_-mediated DNA lesions in sperm affect the first wave of active DNA demethylation that is 5hmC independent. We also want to highlight here that the setting of our experiment in the context of DNA demethylation analysis in the zygote is unique of its kind. Indeed, all the studies so far have used strategies to impair 5hmC (i.e. *Tet3* deletion), whereas the impairment of DNA demethylation was never used since it is not know how this process occurs. Therefore, our study represents the first analysis of 5hmC under conditions where DNA demethylation is impaired.

The equal presence of 5hmC in the maternal pronuclei, which do not undergo a global active demethylation, suggests that some events of pre-replicative TET3-mediated DNA demethylation can also occur in the maternal genome. This result is also consistent with the pre-replicative replacement of unmodified cytosines in the maternal pronuclei detected in this work and with previous studies showing locus-specific active DNA demethylation in the maternal genome [29, 75]. Alternatively, the conversion of 5mC to 5hmC is not implicit to active DNA demethylation but more linked to passive demethylation as previously proposed [63].

Quantification of total 5mC and 5hmC content in bovine gametes revealed another peculiar epigenetic species-specific feature. 5mC and meCpG levels in mouse and human gametes were reported to be higher in the sperm than in the oocytes [9, 29, 80]. In contrast, our measurements in bovine gametes revealed that the oocytes contain higher global 5mC compared with sperm. Thus, the different 5mC content between bovine female and male gametes underlies a novel species-specific epigenetic feature.

In a clinical context, this study has relevance in assisted reproductive techniques (ARTs) that are commonly used in human medicine and livestock breeding. Nowadays, children conceived using ART account for 2% of all births, which has brought the society a growing interest in their long-term health [12]. Moreover, in vitro production of bovine embryos is used worldwide for commercial purposes. Meta-analysis in human and veterinary medicine has correlated increased ROS levels in sperm to a reduction in success when using ARTs [2]. The fact that oxidative stress in sperm induces not only DNA lesions but also epigenetic alterations strongly indicates the importance to identify sub-fertile patients that potentially harbour high levels of oxidative DNA damage within their spermatozoa. Indeed, the use of ARTs with these patients will undoubtedly increase the likelihood that a spermatozoon harbouring genetic/epigenetic lesions will achieve fertilization by bypassing a number of natural selection strategies that would normally be operating in vivo [44]. In vitro production of bovine embryos with frozen/thawed semen is a standard method. Exposure of sperm to high levels of ROS in the sperm freezing/thawing process was indicated as a potential inducer of DNA damage and decrease in sperm fertility [8, 10, 31]. Accordingly, the supplementation of a cryopreservation extender with antioxidant has been shown to provide a cryoprotective effect on mammalian sperm quality [10, 40]. Our work has shown that oxidative stress compromises not only the integrity of sperm DNA but also its post-fertilization epigenetic reprogramming with consequent defects in early embryo development. Considering the increase use of ART in human medicine and livestock breeding in the upcoming years, optimization of the ART procedures and understanding of their effect on epigenetic reprogramming in the early embryo is a necessary step to determine health risks that may be associated with these reproductive technologies.

## Conclusions

In this study, we have shown that oxidative stress in sperm has an impact not only on DNA integrity but also on the dynamics of epigenetic reprogramming, which may harm the paternal genetic and epigenetic contribution to the developing embryo and affect embryo development and embryo quality. The recruitment of the BER component XRRC1 to the paternal genome harbouring oxidative DNA lesions is indicative that the damage is recognized and likely repaired. Previous data implicated BER in the zygotic active DNA demethylation. Our results further supported the proposed role of BER in this process by showing that the presence of oxidative DNA lesions at the paternal genome impairs active DNA demethylation. Remarkably, the lack of any evident changes in 5hmC levels under conditions where DNA demethylation was impaired indicated that the involvement of the cytosine hydroxylation in this process is more complex than previously thought. Finally, this study highlighted striking differences in DNA methylation dynamics between bovine and mouse zygotes. The 5mC loss/5hmC gain of paternal genome appears to be a specific feature of mouse zygotes, whereas the presence of 5hmC at both parental genomes in bovine embryos is very similar to what has been reported in human embryos. Our results proposed the bovine model as a system to facilitate the understanding of the dynamics of DNA methylation in early development.

## Methods

### Sperm chromatin structure assay

Cryopreserved semen from a bull with proven fertility from an approved artificial insemination (AI) station was used for subsequent experiments (Besamungsverein Neustadt an der Aisch, Germany). Two 0.25-ml straws containing 15 million sperm cells/ml in an egg yolk extender were thawed in a water bath at 37 °C for 30 s. Separation of the sperm was performed with a gradient centrifugation (600 ×*g* for 15 min) with 90% Percoll (Sigma). The sperm pellet was collected, separated into two groups (untreated and H_2_O_2_) and transferred in H-Talp (1 M NaCl, 32 mM KCl, 4 mM NaH_2_PO_4_·H_2_O, 1U penicillin G Na salt, 250 mM NaHCO_3_, 250 mM Hepes, 170 mM CaCl_2_, 49 mM MgCl_2_·6H_2_O, 330 mM Na lactate, 33 mM Na pyruvate, 1% (v/v) NEAAs, 2% (v/v) EAAs). Sperm in the H_2_O_2_ group were treated with 100 μM H_2_O_2_ (Sigma) for 1 h at 37 °C prior to IVF. The control group (untreated) was kept in H-Talp without H_2_O_2_. Samples were centrifuged (600×*g* for 3 min) and washed several times with H-Talp to eliminate H_2_O_2_ traces.

Sperm chromatin structure assay (SCSA™) was performed by diluting sperm samples to a concentration of 2 × 10^6^ sperm/mL with TNE buffer (0.01 M Tris–HCl, pH 7.4, 0.15 M NaCl, 1 mM EDTA). 200 µl of the sperm suspension was mixed with 400 μl of an acid-detergent solution (pH 1.2, 0.08 N HCl, 0.15 M NaCl, 0.1% Triton X-100) and vortexed for 30 s. 1.2 ml of acridine orange solution (6 mg/mL) in a phosphate–citrate buffer (0.2 M Na_2_HPO_4_, 0.1 M citric acid, 0.15 M NaCl, 1 mM EDTA, pH 6.0) was added to the sample and incubated for 3 min. Sperm chromatin structure was measured using an Epics XL-MCL flow cytometer (Beckman Coulter, Fullerton, CA, USA). Cells were exposed to a laser beam generated by a 488-nm argon laser (Laser Components, Olching, Germany). Fluorescence detectors 1 and 3 (FL1 and FL3) were used for detection of green (515–530 nm) and red fluorescence (> 630 nm), respectively. Flow cytometric data were acquired using EXPO 32 ADC XL4 Colour software (Beckman Coulter, Fullerton, CA, USA). Ten thousand events were counted per sample with a flow rate of 200–400 events per sec. Debris, which are non-sperm events, were gated out based on the forward scatter and sideward scatter dot plot by drawing a region enclosing the cell population of interest. The number of sperm showing a high degree of DNA fragmentation was determined by SCSA™ [21]. Cell gating and quantification of the percentage of sperm cells with high DNA fragmentation index (%DFI) was performed as previously described [21] and analysed using FCS express 4 FLOW research edition (De Novo Software, Glendale, USA).

### Computer-assisted sperm analysis (CASA)

To assess sperm motility and morphological analysis, we used the Hamilton Thorne IVOS II CASA driven by the software version 14 (Hamilton Thorne Research, Beverly, MA, USA) according to the manufacturer’s guidelines. The sperm were Percoll centrifuged and H_2_O_2_ treated the same way as for the SCSA™. Six μl of semen was placed in a chamber of a Leja 20-mm two-chamber slide (Leja Products BV, Nieuw Vennep, Netherlands). The percentage of progressive motile sperm at 37 °C was assessed in a minimum of 1000 cells in no less than eight randomly selected fields, with 30 frames acquired per field at a frame rate of 60 Hz.

### In vitro production of bovine embryos

Bovine ovaries were retrieved from the nearby slaughterhouse and transported in 0.9% NaCl (Braun) at 38 °C within 2 h. Cumulus–oocyte complexes (COCs) were isolated using the slicing method from Eckert and Niemann [19]. Under a stereomicroscope with a warming plate at 38 °C, COCs with several layers of compact cumulus cells and a homogeneous cytoplasm were selected and transferred to holding medium (Hepes-buffered TCM-199 supplemented with 10% (v/v) foetal bovine serum).

In vitro maturation (IVM) was performed by grouping 10 COCs in 50 µl microdroplets covered with oil in BO-IVM medium (IVF Bioscience) for 18 to 22 h with 5% CO_2_ in air, saturated humidity and 38.2 °C.

Sperm were treated with H_2_O_2_ as described above. The sperm pellet was transferred into 100 μl BO-IVF medium (IVF Bioscience) and centrifuged for 3 min at 600×*g*. Twenty COCs were transferred into a 100-μl droplet of BO-IVF medium under mineral oil. Sperm samples were added to the IVF droplets to obtain a final concentration of 2 × 10^6^ sperm/ml, and cultures were performed with 5% CO_2_ in air and saturated humidity at 38.2 °C.

Presumptive zygotes were denuded from their cumulus cells 24 h after IVF using a capillary (Stripper, 135 μm, Origio) and transferred in groups of 10–40 µl microdroplets of BO-IVC (IVF Bioscience) under mineral oil (Sigma). 48 h after IVF, the cleavage rate was evaluated and the cleaved embryos were separated from the non-cleaved ones and further cultured with 5% CO_2_, 5% O_2_ at 38.2 °C and saturated humidity.

### Fluorescence microscopy and image analysis

Bovine zygotes were collected 20 h post-in vitro fertilization (pIVF) and incubated in 0.1% hyaluronidase solution (w/v) (Sigma) followed by the denudation with a capillary (Stripper, 135 μm, Origio). The zona pellucida was removed by incubating zygotes with pronase (Sigma, 5 mg/ml in H_2_O) for 3 min at 37 °C. Zygotes were washed three times with PBS buffer (Sigma), fixed with 4% paraformaldehyde (PFA) for 1 h at room temperature (RT) and washed three times with PBS buffer. Permeabilization was performed by incubating zygotes with 0.5% Triton X-100 (Thermo Scientific) in PBS for 15 min at RT followed by three washes with PBS buffer.

5mC and 5hmC immunostaining. After fixation, zygotes were incubated with 4 M HCl for 10 min at RT, followed by a neutralization step with 100 mM Tris–HCl (pH 8) for 10 min. After three times washing with PBS buffer, embryos were incubated with a blocking buffer (3% BSA, in PBS, Sigma) for 1 h at 4 °C followed by incubation with antibodies 5mC (Diagenode/C15200081-100, diluted to 1:5000) or 5hmC (Active Motif/39769, diluted to 1:500) in PBS buffer containing 1.5% BSA and 0.25% Triton X-100) overnight at 4 °C. Samples were washed three times with PBS and incubated with secondary antibodies FITC or Cy3 diluted 1:100 with PBS, 1.5% BSA and 0.25% Triton X-100 for 2 h at RT. After washing three times with PBS buffer, samples were mounted on glass slides in mounting medium (Vectashield containing DAPI, Vector Laboratories) and analysed using an inverted Leica CTR6000 microscope (software: Leica Microsystems LAS-AF6000; Leica Microsystems, Bensheim, Germany).

γH2AX immunostaining. After fixation, embryos were incubated with a blocking buffer (PBS containing 1.5% BSA and 0.25% Triton X-100) for 1 h at 4 °C. Samples were transferred to a 500-μl droplet containing γH2AX antibodies (Biolegend/613402) diluted 1:1000 in 1.5% BSA in PBS buffer for 2 h at RT. Embryos were washed with 0.5% Triton X-100 in PBS for 10 min at RT and incubated with secondary antibodies (FITC mouse, 1:100) overnight at 4 °C. After washing with PBS, samples were mounted on glass slides in mounting medium (Vectashield containing DAPI, Vector Laboratories) and analysed using a Leica microscope.

XRCC1 immunostaining. After fixation, embryos were incubated with a blocking buffer (PBS containing 3% BSA) for 1 h at 4 °C. Samples were transferred to a 40-μl droplet of XRCC1 (XRCC1 Thermo Fisher/MS-1393-P0; dilution 1:500) in 1.5% BSA in PBS and covered with oil. Five µl of 0.5% Triton X-100 was added to the droplet, and samples were incubated for 2 h at room temperature. After washing with PBS, samples were incubated with FITC-conjugated secondary antibodies diluted 1:100 with PBS buffer containing 1.5% BSA and 0.25% Triton X-100 for 2 h at room temperature. After washing with PBS, samples were mounted on glass slides in mounting medium (Vectashield containing DAPI, Vector laboratories) and analysed using a Leica microscope.

Quantification of the immunofluorescence pictures. The images were analysed using ImageJ Software (ImageJ 1.48v, National Institute of Health, USA). Each pronucleus was measured individually. The equal staining of the cytoplasmic area was subtracted (staining background). The microscope settings (exposure time and gain) within each individual experiment remained the same, to ensure comparability. Statistical analysis was performed using GraphPad Prism (Prism for Mac OS X, version 5.0a; two-tailed Student’s *t* test).

### Mass spectrometry

Genomic DNA (gDNA) of 50–150 two-cell embryos or MII oocytes cleaned from cumulus cells was extracted using ZR-Duet DNA/RNA Miniprep kit (Zymo Research) following manufacturer instructions and eluted in LC/MS-grade water. MII oocytes were obtained by placing the processed oocytes into maturation for 22 h (in accordance with the maturation for the IVF procedure). gDNA extraction of sperm was performed according to a modified protocol from [74]. The sperm were treated with H_2_O_2_ as described above (SCSA), subsequently washed in 750 μl H-Talp and centrifuged for 3 min at 600 x g. After centrifugation, pellet was transferred into a 200-μl lysis buffer (10 mM Tris–HCl pH 8, 10 mM EDTA, 2% SDS and 80 mM DTT) and inverted gently. 100 μg/ml RNase A (Thermo Fisher) was added and incubated overnight at 37 °C. Upon proteinase K digestion (200 μg/ml at 55 °C overnight), gDNA was purified by a phenol/chloroform/isoamyl alcohol extraction, followed by ethanol precipitation. The obtained gDNA was resuspended in 50 μl H_2_O. DNA was digested to nucleosides for a minimum of 9 h at 37 °C using a digestion enzymatic mix (kind gift from NEB). All samples and standard curve points were spiked with a similar amount of isotope-labelled synthetic nucleosides: 50 fmol of dC* and dG* purchased from Silantes, 2.5 fmol of 5mdC* and 250 amol of 5hmdC* obtained from T. Carell (Center for Integrated Protein Science at the Department of Chemistry, Ludwig-Maximilians-Universität München, München, Germany). Standard curves were set up for dC and dG (Berry & ass.) from 5 pmol to 0.1 fmol and for 5mdC (Carbosynth) and 5hmdC (Berry & ass.) from 250 fmol to 5 amol. The nucleosides were separated on an Agilent RRHD Eclipse Plus C18 2.1 × 100 mm 1.8u column by using the HPLC 1290 system (Agilent) and analysed using an Agilent 6490 triple quadrupole mass spectrometer. To calculate the concentrations of individual nucleosides, standard curves representing the ratio of unlabelled and isotope-labelled nucleoside peak responses were generated and used to convert the peak area values to corresponding concentrations. The threshold for peak detection was a signal-to-noise ratio (calculated with a peak-to-peak method) above 10, and the limit of quantification was 25 amol for 5mdC and 5hmdC. Final measurements were normalized by dividing by the dG level measured for the same sample.

### EdU/EdC labelling

IVF medium was supplemented with either EdC (200 M, Sigma-Aldrich/T511307) or EdU (200 M, Invitrogen/C10337). 12 h after fertilization, zygotes were denuded and the zona pellucida was removed. Fixation was performed with 4% PFA for 30 min at room temperature (protected from light). Zygotes were washed with PBS and permeabilized with PBS buffer containing 0.5% Triton X-100 for 15 min at room temperature. Zygotes were washed twice with PBS, transferred into 40 μl droplets of Click-it^®^ reaction cocktail (Invitrogen), covered with oil and incubated for 30 min at room temperature protected from light. Zygotes were washed with PBS and mounted on slides with Vectashield containing DAPI.

### BrdU/EdC labelling

Embryos were fertilized in IVF medium supplemented with nucleotide analogues BrdU (100 μM, Roche/10280879001) and EdC (200 μM, Sigma-Aldrich/T511307). 12 h after IVF, zygotes were denuded and the zona pellucida was removed. Control zygotes were stained 24 h pIVF. Zygotes were incubated with PBS and fixed with 4% PFA for 30 min at room temperature (protected from light). Zygotes were washed with PBS, and permeabilization was performed with 0.5% Triton X-100 in PBS for 15 min at room temperature. Zygotes were washed with PBS followed by denaturation with 3 M HCl for 10 min and a neutralization step with 100 mM Tris–HCl (pH 7.5) for 10 min. After washing, zygotes were transferred into 40 μl droplets of Click-it^®^ reaction cocktail covered with oil and incubated for 50 min at room temperature protected from light. Samples were then washed and incubated with a solution containing anti-BrdU antibody (Roche/11170376001; 6 ng/μl in 1.5% BSA and PBS) for 1 h at RT. Zygotes were washed with PBS, incubated with Cy3-conjugated secondary antibodies for 1 h at RT, washed again with PBS and mounted on slides with Vectashield containing DAPI.

## Additional file

**Additional file 1.** Oxidative stress in sperm does not affect paternal genome decondensation in zygotes. Quantification of the DAPI signal in the paternal and maternal pronuclei in zygotes from control and H_2_O_2_-treated groups.
