# Supplementary material for: Oxidative stress in sperm affects the epigenetic reprogramming in early embryonic development
Source: Epigenetics Chromatin. 2018 Oct 17;11:60. doi: 10.1186/s13072-018-0224-y (PMC6192351; doi:10.1186/s13072-018-0224-y)
Supplement: Supplementary file 1 — Additional file 1. Oxidative stress in sperm does not affect paternal genome decondensation in zygotes. Quantification of the DAPI signal in the paternal and maternal pronuclei in zygotes from control and H2O2-treated groups. [file 13072_2018_224_MOESM1_ESM.pdf]

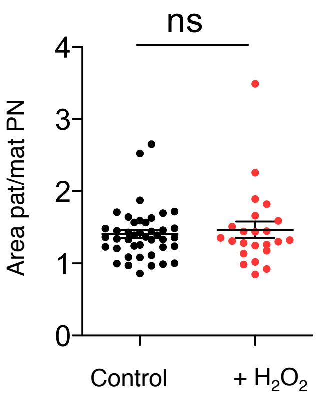

Fig. S1 - Oxidative stress in sperm does not affect paternal genome decondensation in zygotes. Quantification of the DAPI signal in the paternal and maternal pronuclei in zygotes from control and H_2O_2-treated groups.
